# Stereotactic radiotherapy in the treatment of ocular melanoma: A noninvasive eye fixation aid and tracking system

**DOI:** 10.1120/jacmp.v4i2.2531

**Published:** 2003-03-01

**Authors:** S. M. Jaywant, E. K. Osei, S. Ladak

**Affiliations:** ^1^ Department of Radiation Physics Princess Margaret Hospital 610 University Avenue Toronto Ontario M5G 2M9 Canada; ^2^ Department of Radiation Therapy Princess Margaret Hospital 610 University Avenue Toronto Ontario M5G 2M9 Canada

**Keywords:** eye fixation aid, eye‐tracking, ocular melanoma, stereotactic radiotherapy

## Abstract

Ocular melanoma is frequently treated using brachytherapy implants (such as ${}^{125}I$ and ${}^{60}\text{Co}$ plaques or ${}^{184}\text{Ta}$ wire), surgery, or external beam radiotherapy using small ${}^{60}\text{Co}$ beams, high energy x‐rays, or proton therapy. The last technique, though very expensive, provides improved dose distributions and dose localizations in the treatment of tumours adjacent to critical normal tissues. The technique of fractionated stereotactic radiotherapy is now being used at an increasingly large number of centers in the treatment of lesions in the brain, and the head and neck. This article describes the successful extension of the stereotactic technique to the treatment of ocular melanoma: an eye fixation aid is attached to a noninvasive, relocatable Gill‐Thomas‐Cosman head frame together with a simple eye‐movement tracking system.

PACS number(s): 87.53.–j, 87.90.+y

## INTRODUCTION

Conventional radiation therapy in the treatment of intracranial lesions involves considerable portion of healthy brain in the treatment volume. Linear accelerator based fractionated stereotactic radiotherapy, however, combines stereotactic localization with fractionated dose delivery.^1^ ^,^ ^2^ The normal tissue sparing thus provided, allows tumours that are in close proximity to critical structures to be treated conformally and to a high dose. Craniopharyngioma,^3^ glioblastoma, meningioma, and pituitary adenoma are examples of some of the sites that are being successfully treated by stereotactic radiotherapy.

Current strategies available for patients with ocular melanoma include observation, external beam radiation, application of plaques, enucleation, and proton therapy.^4^ ^–^ ^7^ Enucleation is still generally agreed to be the choice of treatment for large tumours.^8^ External beam radiation can be used to treat medium size (i.e., a height of about 2.5 to 10 mm) lesions in the posterior one‐third of the globe of the eye that are not suitable for brachytherapy (e.g., tumours close to the optic disc).^9^ Iodine‐125 plaques, which are now commonly used, are hard to fashion around lesions that are in close proximity to the optic disc and would also deliver a very high dose to the head of the optic nerve. Proton therapy,^10^ ^,^ ^11^ though able to overcome some of these limitations, is a very expensive technique. It has now become possible to treat small to medium size ocular melanomas using stereotactic radiotherapy with acceptable dose distributions and target localizations and should provide a good alternative to proton therapy.

The primary issue in the treatment of ocular melanoma by stereotactic radiotherapy is the reproducibility of the eye position at CT and during each fractionated treatment.^12^ ^,^ ^13^ The treatment planning system always utilizes the CT image set to derive the treatment coordinates (vertical, lateral, and anterior‐posterior) and hence it is of utmost importance that the lesion, with respect to the head frame, be in the same location during treatment delivery as that at the time of acquisition of the CT image set. A simple eye fixation aid that attaches to the head frame is therefore used to help the patient fixate the eye and thus ensure the reproducibility of eye position. In addition to the eye fixation aid, an eye tracking system has been developed for monitoring the reproducibility of the eye position. The latter is achieved by the application of an image‐processing program that performs a pixel‐by‐pixel subtraction of the images taken at CT and during treatment delivery.

A variety of noninvasive immobilization systems are used for fractionated stereotactic radiotherapy.^14^ ^,^ ^15^ At our institution, stereotactic radiotherapy is delivered using the Radionics (TycoHealthcare) system. It consists of the noninvasive, relocatable Gill‐Thomas‐Cosman (GTC) frame and utilizes the couch‐mounted system on a Varian 2100 C/D linear accelerator. This method relies on the use of multiple non‐coplanar converging arcs all intersecting at the intended target. Treatment planning is accomplished using the XKnife‐4 software, and 6 MV x‐rays are used for treatment delivery.

## MATERIALS AND METHODS

### The eye tracking system

**Figure 1:**
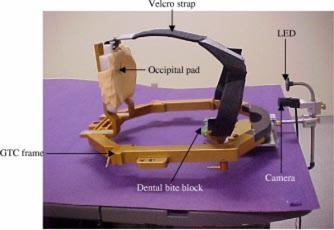
(Color) Patient immobilization system (e.g., a GTC frame and the dental bite block assembly) and the eye tracking system (i.e., the LED and miniature camera).

The eye tracking system consists of a fixation aid, a miniature camera, and an image processing software as described below:

(i) *Fixation aid.* The eye fixation aid is to help the patient to focus at a fixed position throughout the CT and treatment. This fixation aid is made up of a right‐angled mounting arm that attaches directly to the bottom of the GTC frame and at the same location as the dental plate. Two alignment pins on the mounting arm mate into the same alignment holes as the alignment pins on the dental plate (see Fig. 1). The attachment screws are required to be longer when coupling both the dental plate and the mounting arm onto the GTC frame. As shown in Fig. 1, a clamp allows a rod (diameter 0.5 cm and length 25 cm) to be fixed to the mounting arm. A second clamp is used to attach a hollow cylindrical tube to the rod.

The hollow cylindrical tube (diameter 0.6 cm and length 8 cm) houses a green light emitting diode (LED) with the power cable emerging from the opposite (rear) end of the tube. A black delrin disc with a 0.4 cm aperture is attached to the front end of this tube. This allows the patient to easily view the center of the illuminated LED against a black background. The LED itself is powered by a 3‐V battery pack placed in a separate box. A potentiometer is used to vary the intensity of the LED since each patient may have different comfort levels.

**Figure 2:**
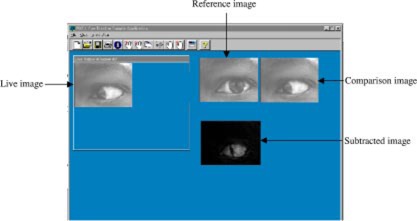
(Color) A typical screen display showing the positions of the live, reference, comparison, and subtracted images.

As shown in Fig. 1, the first clamp provides a vertical adjustment as well as rotation of the entire LED assembly with respect to the mounting arm/dental plate in two orthogonal planes. The second clamp gives additional flexibility in adjusting the height and angle of the LED with respect to the mounting arm. However, it is most useful in setting the distance of the LED from the eye.

(ii) *Imaging system.* The imaging system consists of a miniature camera and an image processing software that we have developed. The miniature camera is attached to the cylindrical tube (housing the LED) under the delrin disc (Fig. 1). The position of the camera can be adjusted in the vertical direction and can be focused on one or both eyes of the patient. The camera is connected via a frame grabber to a computer that has the image processing software. A typical screen display on the computer has an active window that shows images of the eye in real time, the reference image, the comparison, and subtracted images (Fig. 2). The reference image is an image that is captured during the CT scan, and it specifies the eye position at the time when it is being scanned. All images obtained during treatment delivery, called comparison images, are compared to this reference image. The subtracted images then are the differences between the reference and comparison images and are displayed on a gray scale from 0 (black) to 255 (white). A completely black subtracted image indicates no shift in the eye position during treatment as compared to the reference obtained at CT.

For a typical ocular melanoma patient, once a patient‐specific dental impression and an occipital support pad are made the GTC frame is assembled. The eye fixation aid is then attached to the GTC frame as described earlier. Adjustments are made to the fixation aid so that the LED is positioned in front of the eye not being treated. It is ensured with the help of the eye clinic that the two eyes track together and are normal with respect to fixation and movements. A check is made to ensure that the additional device does not obstruct the quality assurance hardware routinely used in stereotactic radiotherapy. At this point the various clamps are tightened and reference marks made on the device to highlight the positions of the various components with respect to each other. This serves as a way of checking before every treatment that there has been no accidental shift in the positions of the components. The whole setup is now patient specific.

During the CT scan acquisition, the patient is asked to focus on the LED when scanning through the eye. This then determines the target position and subsequent treatment planning using the XKnife software. Also during the scan a reference image is captured for future comparisons with images captured during treatment. At the time of treatment delivery the patient is asked to fixate the eye on the LED. Real time images of the eye can be observed on the computer screen and, at equal intervals of time, comparison images are captured. These images are compared to the reference image captured during CT (by doing a pixel‐by‐pixel subtraction of the comparison from the reference image). The subtracted image gives an indication of any eye movement from the reference position at CT. This process of acquiring comparison images and viewing the subtracted images online at short intervals during the beam on allows the radiation therapist to track any motion of the eye and abort treatment if necessary. All the images are saved and also can be analyzed at the end of the day.

## DISCUSSION AND CONCLUSION

**Figure 3:**
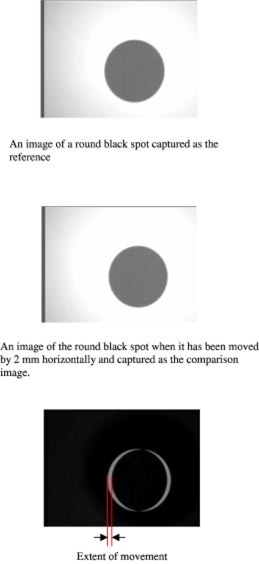
(Color) (a) An image of a round black spot captured as the reference. (b) An image of the round black spot when it has been moved by 2 mm horizontally and captured as the comparison image. (c) Subtracted image between the reference and comparison images. The width of the white patches (arrowed) indicates the extent of object movement.

**Figure 4:**
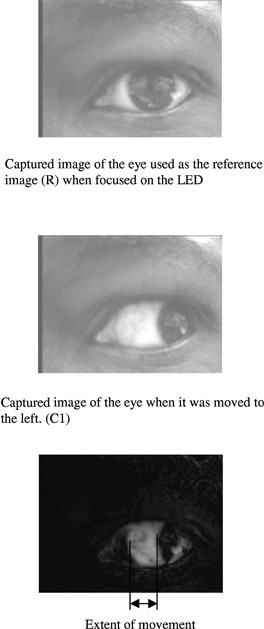
(a) Captured image of the eye used as the reference image (R) when focused on the LED. (b) Captured image of the eye when it was moved to the left (C1). (c) Subtracted image (R‐C1) showing the extent of eye movement to the left.

The utility of the software is demonstrated in Fig. 3, wherein an image of a black disc in a given position is considered as the reference image [Fig. 3(a)]. The object is then moved horizontally by 2 mm and a comparison image is captured as shown in Fig. 3(b). The subtracted image, which represents the difference between the reference and the comparison images, is shown in Fig. 3(c). The software is able to detect the object movement and displays it as white area on the subtracted image. The system can detect movements of about 0.3 mm. The ability of the software to detect movement was again tested using the eye. In Fig. 4(a), an image of the eye was captured (as reference) when it was fixated on the LED. The eye was then moved to the extreme left and a comparison image captured [Fig. 4(b)]. The subtracted image [Fig. 4(c)] shows a maximal eye deviation of about 9 mm, and it is indicated by the white area on the image.

Typical fractionation schedule for ocular melanoma patients is 70 Gy in five fractions over a period of 10 days with 4 or 5 arcs/beams in XKnife. This implies that the beam on time is quite large and resembles that in a radiosurgery. In addition, ocular melanoma patients generally tend to fall in the older age group, typically 55 to 65 years, which can make it difficult for them to focus at the LED continuously for the duration of the beam on. Thus, eye tracking is essential when treating ocular melanoma by the stereotactic radiotherapy technique. Although this system has not yet been used on patients, it has the potential of being clinically useful for ocular melanoma patients. Future developments could include linking the tracking system with the gating of the linac. This would pause the beam if an eye motion was detected that was outside the tolerance level and resume once the eye position is restored.
